# Account of Diseases at Bristol and in London

**Published:** 1801-11

**Authors:** 


### *To the Editors of the Medical and Physical Journal.*

GENTLEMEN,

THE state of the patients admitted on the Bristol Dispensary, from the 1st of September to the 1st of October, 1801, is as follows:

Anasarca

| | | | |
|---|---|---|---|
| Anaſarca | 3 | Fits | 1 |
| Amenorrhœa | 1 | Impoſtume | 1 |
| Aſthenia | 2 | Nervous | 1 |
| Aſthma | 1 | Pleuritis | 2 |
| Abſceſs in the Axilla | 1 | Phthiſis | 1 |
| Contuſion | 1 | Peripneumonia | 2 |
| Conſumption | 1 | Rheumatiſmus Acutus | 3 |
| Diarrhœa | 14 | Rheumatiſmus Chronicus | 6 |
| Dyſenteria | 22 | Synocha | 8 |
| Diſeaſed Breaſt | 1 | Scrophula | 1 |
| Eryſipelas | 2 | Typhus Fever | 39 |

*Diſpenſary House, Oct.* 9, 1801.

